# Early Hydration Heat of Calcium Sulfoaluminate Cement with Influences of Supplementary Cementitious Materials and Water to Binder Ratio

**DOI:** 10.3390/ma14030642

**Published:** 2021-01-30

**Authors:** Jun Zhang, Guoju Ke, Yuzhang Liu

**Affiliations:** 1 Key Laboratory of Safety and Durability of Civil Engineering, China Education Ministry, Beijing 100084, China; 2 Department of Civil Engineering, Tsinghua University, Beijing 100084, China; keguoju@mails.tsinghua.edu.cn (G.K.); liuyuzhang@mails.tsinghua.edu.cn (Y.L.)

**Keywords:** calcium sulfoaluminate cement, hydration heat, supplementary cementitious materials, water to cement (binder) ratio

## Abstract

Compared to ordinary Portland cement (OPC), calcium sulfoaluminate cement (CSA) displays very early-age strength and faster heat-releasing rate during hydration. In the present paper, the early hydration heat of CSA paste with influences of supplementary cementitious materials (SCMs) and water to cement (or binder) ratio (w/c) is systematically studied by measuring the heat-releasing rate using a calorimeter. Three traditional SCMs—silica fume (SF), fly ash (FA) and ground granulated blast furnace slag (SL)—were used in the study. A water to cement or binder ratio (w/c) between 0.19 and 0.73 was used in the mixtures. The results show that three exothermic peaks were presented during hydration—dissolution exothermic peak and two reaction exothermic peaks. With the w/c of 0.3, the first and second reaction peaks of the CSA paste are as high as 17.8 times and 4.1 times that of OPC paste, and the occurring time is much earlier than that of the OPC paste. The second reaction peak appears earlier, and the third reaction peak appears later in the pastes with addition of SF than in those without SF. Decreasing w/c can greatly reduce the two reaction peaks of the paste, and it looks that there is a critical value of w/c between 0.24 and 0.30. Above the critical value, the effect of w/c is minor, and below that the influence is obvious. An optimal use of SCMs in CSA pastes under different w/c can greatly decrease the heat releasing while maintaining the required strength.

## 1. Introduction

Calcium sulfoaluminate cement (CSA) has lower CO_2_ emission and firing temperature during production compared with ordinary Portland cement (OPC) [1]. In addition, recent studies have revealed that concrete made with CSA displays relatively low shrinkage, especially drying shrinkage, compared to OPC concrete [2,3,4]. CSA may be chosen as one of the most promising low CO_2_ alternatives to OPC in view of the sustainable development and long-term durability of modern concrete [5,6,7,8,9,10]. The hydration heat generated during the hydration of cement is a basic property of cement-based materials. However, the heat-releasing rate and the cumulative heat of different kinds of cement may vary very much, which may greatly influence the mechanical properties of the material. A number of existing studies have shown that a higher releasing heat of cement hydration will adversely affect the long-term performance of the structures [11,12,13,14]. Typically, a higher temperature rise in concrete structures may result in early-age cracking. In addition, a higher temperature rise in large-volume concrete structures may also lead to the potential of delayed expansion of ettringite (AFt) [15,16,17,18].

The main hydration products of CSA are ettringite (AFt) and Al(OH)_3_. In the case of sufficient gypsum, the formation rate of AFt in the CSA paste is fast, and a large amount of heat is produced during hydration [19,20,21,22]. Therefore, slowing down the hydration rate of the CSA paste and reducing the hydration heat are priorities for the application of CSA cement in concrete structures. For OPC concrete, previous studies have shown that the addition of supplementary cementitious materials (SCMs) in concrete will reduce its early-age strength and increase the strength at a later age. What’s more, the workability and long-term durability of concrete have been greatly improved as well. The use of SCMs can decrease the amount of cement, save costs, reduce hydration heat, and further improve the greenness of the products. For CSA concrete, due to the low content of Ca(OH)_2_ in its hydration products, the pozzolanic effect of SCMs may not be as obvious as in OPC concrete, and they may simply serve as fillers [23,24,25,26]. However, little is known on the effect of different SCMs on the hydration heat of CSA. 

This study aims to study the hydration heat of the CSA paste with the effects of SCMs and water to cement (or binder) ratio (w/c). Three traditional SCMs, silica fume, fly ash and ground granulated blast furnace slag, were used in the study. A total of four series tests were performed in the test programs. The first test series was to study the releasing characteristic of the hydration heat of CSA and OPC with a single water to cement ratio of 0.3. The second test series was designed to study the effect of SCMs on the hydration heat of the CSA paste, in which different combinations of silica fume, fly ash and ground granulated blast furnace slag were used, and a single water to binder ratio of 0.3 was applied. In the third series, the effect of the w/c ratio on the hydration heat of the CSA paste was investigated, and four w/c values (0.24, 0.33, 0.47, and 0.73) were used. The fourth series was designed to study the combined effect of w/b and SCMs on the hydration heat of the CSA paste, in which four pairs of w/c were used. The rate of hydration heat evolution and progress of the accumulative heat of the pastes were measured using a calorimeter. The hydration products at a very early age of the pastes were analyzed by X-ray diffraction (XRD) and thermal decomposition analysis (TG, DTG).

## 2. Experimental Program

### 2.1. Materials

**Figure 1:**
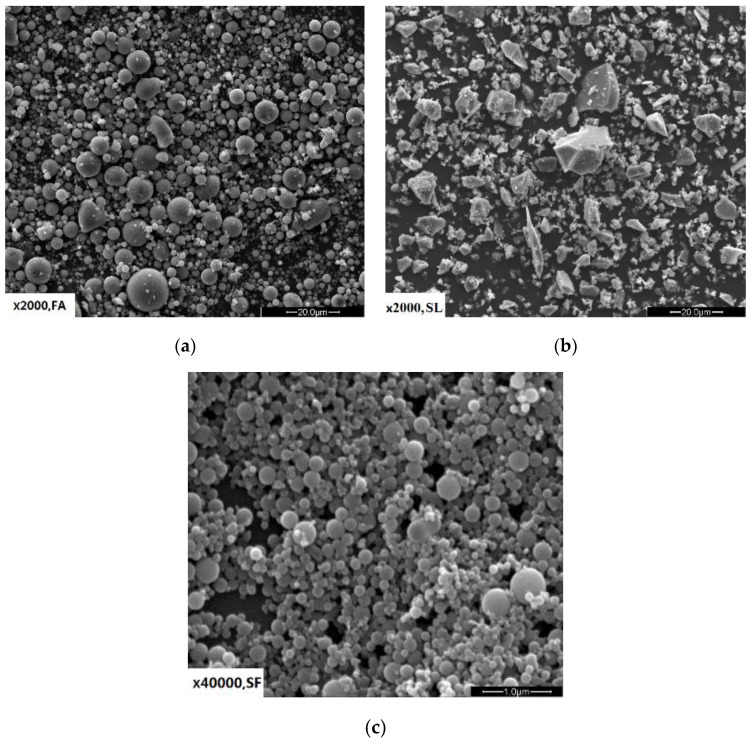
Morphology images of supplementary cementitious materials, (**a**) fly ash, (**b**) slag, and (**c**) silica fume.

**Figure 2:**
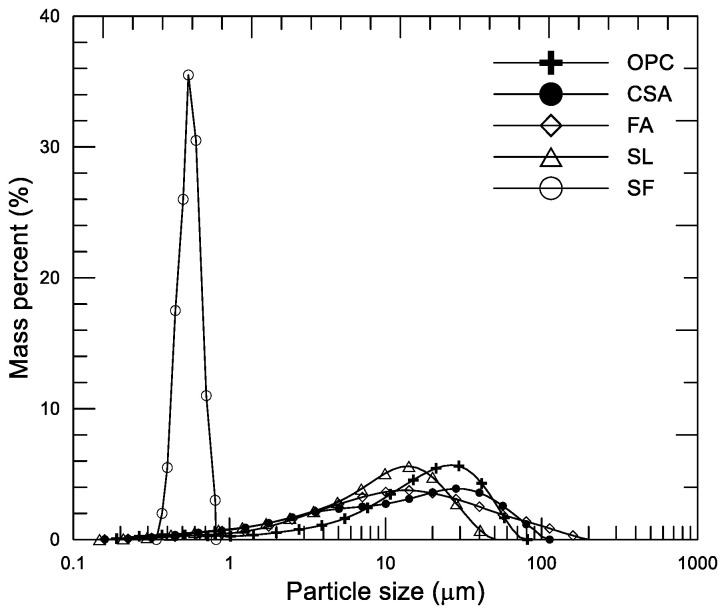
Size distribution of cements and supplementary cementitious materials (SCMs) used in the tests.

The main mineral composition of 4CaO·3Al_2_O_3_·SO_3_ (38.34%), 2CaO·SiO_2_ (31.33%), CaSO_3_ (13.25%), and 12CaO·7Al_2_O_3_ (0.86%) of commercial CSA (grade 42.5) (Beijixiong Building Materials Co. Ltd, Tangshan, China) was used. For comparison, commercial OPC (grade 42.5) (Beijing Jinyu Cement Co. Ltd, Beijing, China) was used in one mixture. The SCMs used in the tests were grade I fly ash (FA), grade S95 granulated blast furnace slag powder (SL), and silica fume (SF) with a silicon content of 90.56%. The chemical compositions and physical properties of the two cements (CSA and OPC) and the three SCMs (SF, FA, and SL) are listed in Table 1. Figure 1 shows the particle morphological characteristic of the three SCMs. Figure 2 shows the particle size distribution of the two cements and the three SCMs. To guarantee the fresh cement pastes had a similar initial fluidity after mixing, a polycarboxylate-based water-reducing agent (PC) was used in the mixtures.

### 2.2. Mixture Proportions

In the test programs, four series of cement paste tests were conducted. The purpose of the first series was to study the releasing characteristic of the hydration heat of CSA and OPC, that includes two mixtures. A water to cement ratio (w/c) of 0.3 was used. Mixture one was OPC paste, labeled as OPC0.3, and mixture two was CSA paste labeled as CSA0.3 (see mixture Nos. 1 and 2). The second test series was designed to study the effect of SCMs on the hydration heat of the CSA paste. In this series, a single water to binder ratio (w/b) of 0.3 was used. Five mixtures were designed, including the reference mixture CSA0.3 (without use of SCMs), 10% FA of the total binder weight (0.9 cement + 0.1 FA) CSA0.3-FA, 10% SF of the total binder weight (0.9 cement + 0.1 SF) CSA0.3-SF, mix use of FA and SF (0.8 cement + 0.1 FA + 0.1 SF) CSA0.3-SF+FA, and mix use of FA, SF, and SL (0.7 cement + 0.1 FA + 0.1 SF + 0.1 SL) CSA0.3-SF+FA+SL (see mixture Nos. 2 to 6). In the third series, the effect of the w/c ratio on the hydration heat of the CSA paste was investigated. In this series, four mixtures were designed, with w/c of 0.24, 0.33, 0.47, and 0.73. These mixtures were labeled as CSA0.24, CSA0.33, CSA0.47, and CSA0.73 (see mixture Nos. 8, 7, 11, and 13). The fourth series was designed to study the combined effect of w/b and SCMs on the hydration heat of the CSA paste. In this series, four pairs of mixtures were formed with w/b from 0.19 to 0.73, in which one mixture was designed with SCMs and the other without. These four pairs of mixtures were CSA0.24 and CSA0.19-SF+FA+SL (w/b = 0.19, 0.24) (see mixture nos. 8 and 9), CSA0.33 and CSA0.3-SF+FA (w/b = 0.3, 0.33) (see mixture Nos. 7 and 5), CSA0.47 and CSA0.42-FA (w/b = 0.42, 0.47) (see mixture nos. 11 and 10), CSA0.73 and CSA0.62-1.5FA (w/b = 0.62, 0.73) (see mixture Nos. 13 and 12). The detailed mixture proportions of all pastes are listed in Table 2.

### 2.3. Test Measurements

The fluidity of the cement paste was measured by a small slump cone with a top diameter of 36 mm, bottom diameter of 60 mm, and height of 60 mm. In the preparation of fresh paste, water and superplasticizer were first added into the mixer, and then cementitious materials were gradually introduced over a time period of 90 s into the mixer at 62 r/min. After 15 s interval, mixing was resumed for an additional 75 s at 125 r/min. Subsequently, the freshly mixed paste was subject to the initial fluidity test. The paste was poured into the cone right away, and then the cone was quickly lifted up. As the flowing of the paste was stopped, the average value of the two spread diameters (SD) perpendicularly crossing the flowed cement paste was recorded as the fluidity of the paste. The early hydration heat test of the cement paste was carried out by a TAM Air Calorimeter (TA Insments-Waters LLC, New Castle, DE, USA) at a constant temperature of 25 °C. The device, whose test accuracy was 0.1 °C, guaranteed an adiabatic state by circulating air, and recorded the temperature changes. After 3 days standard curing, the samples were taken out to stop hydration in anhydrous alcohol, dried at 40 °C to a constant mass, and ground to below 0.08 mm for XRD and thermal decomposition analysis (thermogravimetry, TG, and derivative thermogravimetry, DTG). The mixtures used for XRD and TG-DTG tests are Nos. 1–6. The equipment used for X-ray diffraction analysis is made by Japan (Rigaku International Corporation, Tokyo, Japan), using a Cu target, a wavelength of 0.154 nm, an operating voltage of 45 kV, a current of 200 mA, and a diffraction angle of 2θ from 5° to 70°. A thermal decomposition test on the hydration products of the pastes was performed with a Netzsch STA 449C thermal analyzer (Netzsch Group, Selb, Germany), with a sample heating range of 30–900 °C and a heating rate of 10.0 K/min. The compressive strength at 3 and 28 days after casting was measured. All tests were conducted in a room with a constant temperature of 25 °C. The measured spread diameter (SD) of the fresh paste and compressive strength at 3 and 28 days of the hardened paste are listed in Table 2.

## 3. Results and Discussion

### 3.1. Hydration Products of CSA with SCMs in Early-Age

**Figure 3:**
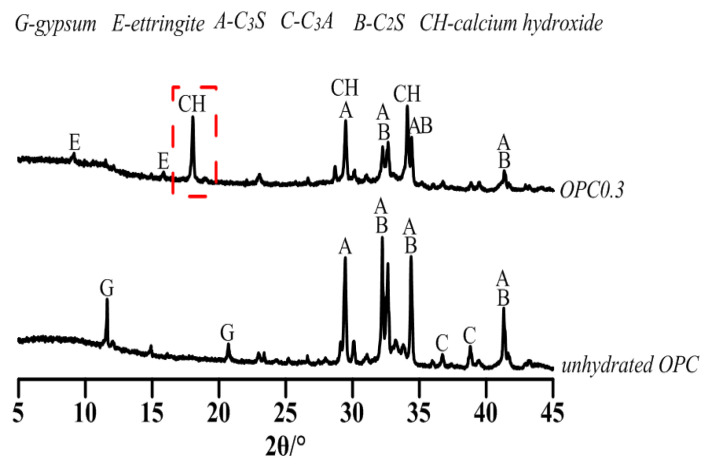
XRD results of OPC paste at 3 days.

**Figure 4:**
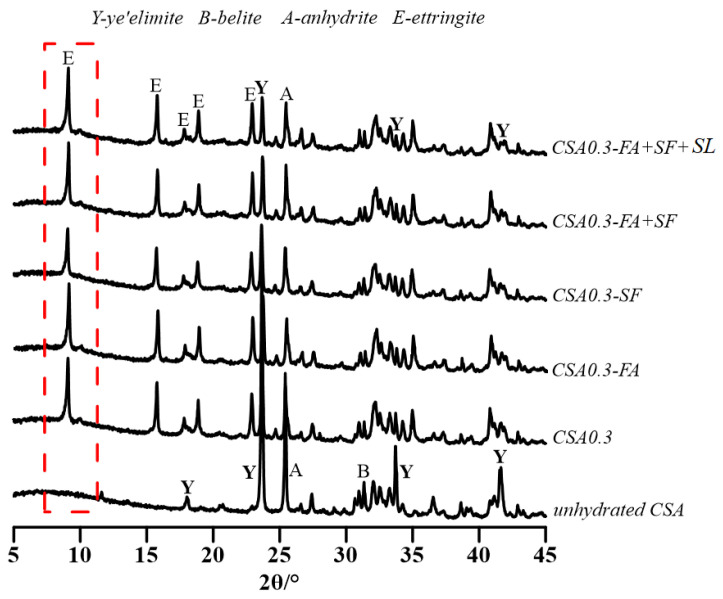
XRD results of calcium sulfoaluminate cement (CSA) paste with SCMs at 3 days.

**Figure 5:**
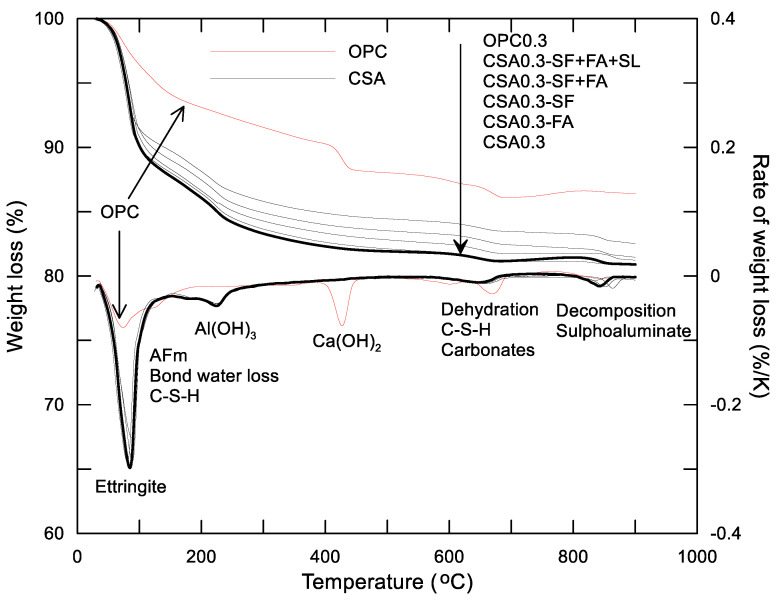
Thermal decomposition analysis (TG-DTG) results of ordinary Portland cement (OPC) and CSA pastes at 3 days.

In order to identify the crystalline phases in the hydration products of the CSA paste and, further, to analyze the effect of SCMs on the hydration of CSA and compare with OPC, the cement paste of mixtures Nos. 1 to 6 in Table 2 were tested at 3 days after mixing by XRD and TG-DTG. The results are shown in Figure 3, Figure 4 and Figure 5.

As can be observed in Figure 3, the main crystalline phases in the hydration products of the OPC paste at 3 days are portlandite and ettringite. The content of minerals (alite, belite, and tricalcium aluminate) is reduced according to their XRD peaks before and after hydration. Therefore, as expected, the hydration products of the OPC paste at this age should be ettringite and portlandite, as well as C-S-H gel, that is confirmed indirectly by the existence of portlandite and the content reduction of alite. The test result showed in Figure 5 also indicates that the hydration products of the OPC paste at 3 days are ettringite, portlandite, and C-S-H gel, for which the temperature at the dewatering rate peak is about 80 °C, 430 °C, and 690 °C, respectively. For CSA paste, the crystalline phase in the hydration products is ettringite after 3-days hydration (see Figure 4). No portlandite was observed. This means that the belite contained in the cement had not reacted with water up to this moment. From Figure 4, a similar intensity of belite is observed before and after the hydration of the cement, which further confirms that no reaction is taken place of belite within the initial 3 days. The delayed hydration of belite was also reported in the literature [27,28]. From the TG-DTG test results of the CSA pastes (see Figure 5), three obvious dewatering rate peaks are observed within 1000 °C. The first dewatering rate peak occurs at about 80 °C and should result from the dewatering of ettringite, which can also be observed in the OPC paste, while the weight loss rate of the CSA paste is much higher than that of the OPC paste. The second obvious dewatering rate peak occurs at about 250 °C and should result from the decomposing of aluminium hydroxide, Al(OH)_3_. No dewatering rate peak is found in OPC paste. It is well understood that the formation of ettringite should accompany the production of the Al(OH)_3_ of the CSA paste [29]. At 430 °C and 690 °C, no dewatering rate peak is observed on CSA paste, which indicates no C-S-H gel and Ca(OH)_2_ in the hydration products at this moment. At about 850 °C, a dewatering rate peak occurs for CSA paste that may be responsible for the decomposition of sulfoaluminate, including monosulfate (AFm) that may be formed by thermal decomposition and by chemical reaction in the stage of ion diffusion controlled hydration of the CSA paste [30]. Therefore, the main hydration products of the CSA paste at this age (3 days) should be AFt, Al(OH)_3_, and AFm. The effect of SCMs on the hydration products of the CSA paste at 3 days is negligible. At 3 days, under the same w/c and given temperature, the weight loss of the CSA paste is obviously higher than that of the OPC paste. The weight loss at 3 days of the CSA paste with different addition of SCMs obeys the following order: CSA0.3 > CSA0.3-FA > CSA0.3-SF > CSA0.3-(FA+SF) > CSA0.3-(FA+SF+SL). It should be noted that the mass loss of different binders, including SCMs, which is governed by their initial mineral composition, should influence the test results of TG-DTG. Therefore, it may not simply give the conclusion that the more weight loss, the more hydration products or the higher the cement hydration degree.

### 3.2. Early Hydration Heat of the CSA Paste with the Effects of SCMs and w/c

#### 3.2.1. Early Hydration Heat of CSA and OPC Pastes with w/c = 0.3

**Figure 6:**
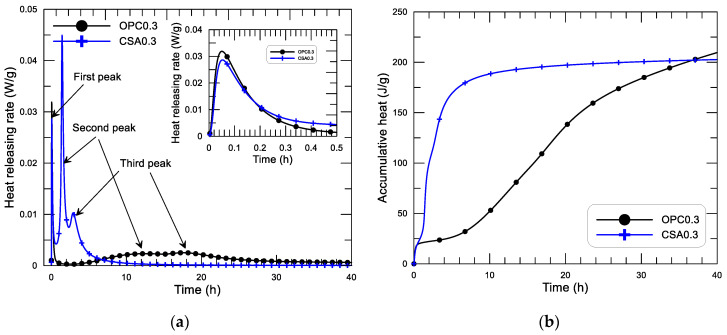
Diagram of releasing rate of hydration heat (**a**), accumulative heat (**b**), and age of CSA and OPC paste with w/c of 0.3.

The test measuring the relationship between heat-releasing rate and mixing time within 40 h since water added of CSA and OPC paste with w/c of 0.3 is displayed in Figure 6a. The corresponding diagram of accumulative heat and age of the two pastes is presented in Figure 6b.

From Figure 6a, three exothermic peaks are observed within the 40 h since the mixing of both the OPC and CSA pastes, although the individual peak strength and occurring time are obviously different between the two pastes. The first peak (see the small inserted figure in Figure 6a) is similar in peak strength and in ascending-descending performance for the two pastes. The second and the third peaks of the CSA paste are much stronger and occur much earlier than that of the OPC paste. As discussed in existing studies on this topic [24,27], the first peak corresponds to the dissolution heat of solid grains that happens immediately after mixing the binder and water. The peak of the releasing rate of the dissolution heat of the OPC paste is a little higher than that of the CSA paste (0.032 and 0.028 W/g, respectively, for OPC and CSA paste), while CSA paste displays a slow decaying rate after the peak. This small difference may be principally due to the differences in the composition of the two cements, especially the dissolution of C_3_A and the possible precipitation of ettringite in OPC paste. The listing period of the dissolution heat releasing of the two pastes is very short (≈0.25 h). This is the so-called the initial hydration period of cement hydration. In the initial hydration period, the total releasing heat of the two pastes is almost the same, 18–20 J/g (see Figure 6b). After that, both of them enter the induction period of cement hydration. The induction period of the OPC paste is relatively longer than that of the CSA paste. Until about 5 h after mixing, the heat flow of the OPC paste starts to grow again, and the hydration process enters the acceleration period. In this period, two heat flow peaks of the OPC paste are observed. One appears at 12.6 h, with a value of 2.34 mW/g, and the other appears at 17.7 h, with a value of 2.53 mW/g. The study by Jansen et al. [27,28] revealed that the accelerative hydration of the OPC paste in the acceleration period is primarily due to the hydration of C_3_S (silicate reaction) and C_3_A (aluminate reaction). The first peak of heat flow in this period is mainly attributed to the silicate reaction, including the dissolution of the alite and precipitation of portlandite and C-S-H gel. The second heat flow peak is attributed to the dissolution of C_3_A, the precipitation of ettringite, and the silicate reaction. For the CSA paste, the induction period is considerably shorter than that of the OPC paste. At about 50 min after mixing, the heat flow of the CSA paste starts to grow up, and the hydration process enters the acceleration period. In this period, two much stronger heat flow peaks than that of the OPC paste are observed. One appears at 1.5 h, with a value of 44.9 mW/g, and the other appears at 3 h, with a value of 10.3 mW/g. The maximum heat flow of the CSA paste is 17.8 times that of the OPC paste. The first heat flow peak of the CSA paste in this period corresponds to the reaction heat of calcium sulfoaluminate, anhydrite, and water to form ettringite, including the dissolution of the sulfates and the precipitation of ettringite. The second peak corresponds to the reaction heat generated from the local formation of ettringite and monosulfate around the cement grains under the ion diffusion and/or migration controlled state, that happens after the formation of a solid frame linking the solid particles. A more detailed test observation and discussion regarding the hydration process of the CSA paste can be found in the literature [23,29,30]. After the second peak, the heat flow decreases with age and the hydration process enters the retardation period.

It can be seen that for the same w/c, the second and third heat flow peaks (both reaction peaks) between the CSA and OPC pastes is obviously different both in the peak strength and in the occurring time. The CSA paste displays a stronger and earlier heat-releasing peak of cement hydration (the second and third peaks) compared to that of the OPC paste. These great differences in the hydration heat-releasing result are obvious different performances of accumulative heat development, as displayed in Figure 6b. From the figure, it can be seen, first, that the dissolution heat resulting from the initial hydration period of the two pastes is almost the same. After that, the CSA paste displays a shorter induction period (≈0.8 h), while the OPC paste presents a longer induction period (≈5 h). In the accelerative period, the CSA paste presents a much faster development of accumulative heat compared with that of the OPC paste. The inflection point of accumulative heat of the CSA paste is reached about 6 h after mixing, while the accumulative heat continuously increases with age at the end of the test, at 40 h. At 36.8 h, the accumulative heat of the OPC paste exceeds that of the CSA paste. The fast hydration and high early-age heat-releasing characteristics of CSA are clearly presented compared with OPC.

#### 3.2.2. Early Hydration Heat of the CSA Paste with Different SCMs

**Figure 7:**
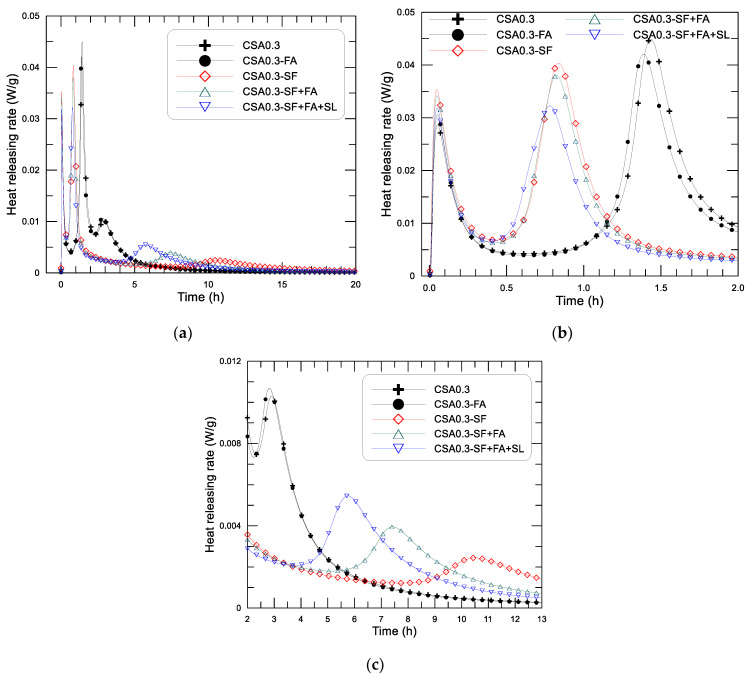
Heat-releasing rate of CSA pastes with different addition of SCMs: (**a**) results of 20 h, (**b**) results of the initial 2 h, (**c**) results of 2–13 h.

**Figure 8:**
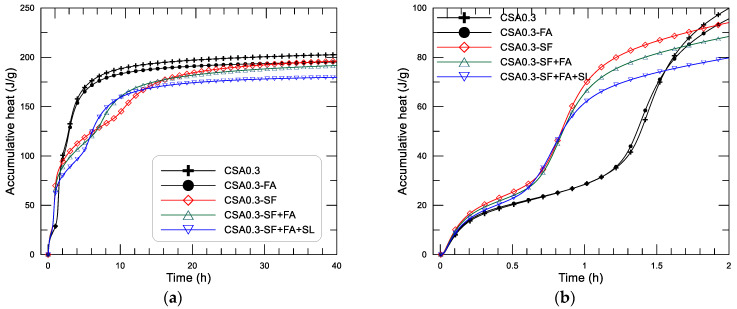
Accumulative heat and age diagram of the CSA paste with different addition of SCMs: (**a**) results within 40 h, (**b**) results within the initial 2 h.

Figure 7 displays the test-determined diagram of hydration-heat-releasing rate and age since the mixing of the CSA pastes (w/c = 0.3) with different addition of SCMs, in which (a) shows the results within 20 h, (b) and (c) are the results within the initial 2 h and the results between 2–13 h to show the variation of the rate peaks more clearly. The corresponding accumulative heat and age diagrams of the pastes are presented in Figure 8, in which (a) shows the results within 40 h and (b) shows the results within the initial 2 h.

From Figure 7, the hydration heat-releasing characteristics presented as three heat flow peaks in the early age can still be observed on the CSA pastes with different use of SCMs. The heat-releasing performance of the CSA pastes (w/c = 0.3) with different addition of SCMs can obviously be divided into two kinds according to whether the mixture contains silica fume. For the mixtures with silica fume addition, such as CSA0.3-SF, CSA0.3-SF+FA, and CSA0.3-SF+FA+SL, the second heat flow peak clearly occurs earlier (at around 0.8–0.9 h) than that of the rest mixtures without silica fume addition, such as CSA0.3 and CSA0.3-FA (at around 1.4–1.6 h) (see Figure 7b). In other words, the addition of silica fume can greatly reduce the length of the induction period of the paste. On the first heat flow maxima (first peak), i.e., the dissolution heat resulted peak, the appearing time of the five mixtures is quite similar, immediately after mixing. But the peak strength is different, ranging from 0.028 to 0.035 W/g. The order of the first exothermic peak is as follows: CSA0.3-SF > CSA0.3-SF+FA > CSA0.3-SF+FA+SL > CSA0.3+FA > CSA0.3. Clearly, the addition of finer particles to the mixture, especially silica fume, can increase the releasing strength of the dissolution heat of the paste. Under the same binder weight, the more the finer particles, the higher the releasing rate of the dissolution heat. These results are understandable because the dissolution heat is normally proportional to the specific area of the solid particles in the system. In addition, the dilution effect of SCMs, especially the ultra-fine silica fume in the binder system, may also improve the dispersion of CSA in water, which will increase the dissolution heat as well [22,23].

Now looking at the second exothermic peak (first reaction peak, see Figure 7b), the nucleation effect of SF on the formation of Aft in the paste significantly shifts the peak to an earlier date, from 1.5 h (CSA0.3, CSA0.3-FA) to 0.8–0.9 h (CSA0.3-SF, CSA0.3-SF+FA, CSA0.3-SF+FA+SL). By contrast, the effect of FA and SL on the formation age of AFt is minor, although FA and SL are finer than the cement grains and certain nucleation action can still be observed—for example, the order of occurring time of the second peak of the pastes with SF and the combination use of SF+FA and SF+FA+SL are as follows: CSA0.3-SF+FA+SL < CSA0.3-SF+FA < CSA0.3-SF. The peak value, on the other hand, decreases with the increase of SCMs, from 10% to 30% of the total binder weight. The value of the second peak is 0.045, 0.042, 0.041, 0.038, and 0.032 W/g, respectively, for CSA0.3, CSA0.3-FA, CSA0.3-SF, CSA0.3-SF+FA, and CSA0.3-SF+FA+SL. It seems that the effect of SF on hydration heat evolution is purely physical, rather than chemical, because no new hydration products are founded after adding it to the mixture at 3 days after mixing, as described in the previous section. However, the addition of SF significantly advances the formation of AFt in the paste, which may influence the setting and strength development of the paste.

The third exothermic peak (the second reaction peak), as displayed in Figure 7c, is much weaker than the second exothermic peak of the corresponding paste, that is, about 1/4 for CSA0.3 and CSA0.3-FA, and about 1/8 for the rest mixtures of the second peak. The magnitude of the third peak is 0.0105 and 0.0102 W/g for CSA0.3 and CSA0.3-FA (group I), and 0.0055, 0.0040, and 0.0030 W/g for CSA0.3-SF+FA+SL, CSA0.3-SF+FA, and CSA0.3-SF (group II), respectively. The appearing time is contrary to that of the second peak, i.e., the appearing time of CSA0.3 and CSA0.3-FA (2.8, 3.0 h) is earlier than that of CSA0.3-SF+FA+SL (5.8 h) CSA0.3-SF+FA (7.5 h) and CSA0.3-SF (10.5 h). The second and third peaks are closer for CSA0.3 and CSA0.3-FA than those of the rest mixtures. Among the mixtures with the addition of SF, the mixture for the latest and the earliest appearance of the third peak is CSA0.3-SF and CSA0.3-SF+FA+SL, respectively. Referencing the hydration analyses on CSA paste carried out by Zhang et al. [29], the third exothermic peak corresponds to the local formation of AFt and/or AFm inside of the hydration product layer around cement grains formed at the second peak under an ion diffusion controlled environment. Owing to the addition of very fine particles, such as SF (average particle size less than 1 μm), in the mixtures, the capillary pore of the paste becomes smaller, and the diffusion rate of ion becomes slower. The third peak of the pastes with SF addition becomes weaker and occurs later than that of the paste without SF. It is interesting to note that SF can advance the second exothermic peak (first reaction peak) of the CSA paste. On the other hand, it can delay the third peak (second reaction peak).

The effect of SCMs on the heat-releasing performance of the CSA paste may be summarized as follows. The three heat-releasing peaks can still be observed after adding the different SCMs to the paste. The effect of SCMs on the dissolution heat peak (first peak) is minor, displaying a slight increase on the exothermic peak as finer SCMs are added. The significant influence of SCMs is reflected on the two reaction exothermic peaks, i.e., the second and the third heat-releasing peaks. The addition of silica fume can greatly reduce the length of the induction period of the paste, thus making the second peak appear earlier. The value of the second peak decreases with the increase of SCMs from 10% to 30% of the total binder weight. The effect of SCMs on the third heat peak (second reaction peak) is mainly reflected by the effect of SF. The addition of SF can delay the appearing time of the third peak and reduce the heat flow strength as well. 

These effects of SCMs on the hydration heat-releasing performance result in different behaviors of accumulative heat development of the paste, as displayed in Figure 8. From Figure 8, it can be seen that the dissolution heat produced in the initial hydration period of the pastes varied between 20.2 J/g and 25.3 J/g for the pastes with different addition of SCMs. As expected, CSA0.3-SF has the highest dissolution heat, and CSA0.3 has the lowest (see Figure 8b). After that, all the pastes undergo an induction period, while the mixtures with SF addition—CSA0.3-SF, CSA0.3-SF+FA, and CSA0.3-SF+FA+SL—are the first to enter the acceleration period (second heat flow peak), at about 0.7 h, with a fast increase of accumulative heat. The rest mixtures (without use of SF)—CSA0.3 and CSA0.3-FA—enter the acceleration stage at about 1.3 h after mixing. After that, mixtures of CSA0.3 and CSA0.3-FA generate the third exothermic peak at an age closer to their second peak, and the accumulative heat continues to increase until the heat-releasing rate is close to zero. The mixtures with SF addition generate the third exothermic peak with a lower rate and a later age compared to their second peak, as displayed in Figure 7c, in which the mixture with only SF (CSA0.3-SF) becomes the weakest and farthest one of the third exothermic peak among the mixtures. Therefore, after the second peak, the accumulative heat of CSA0.3-SF starts to increase again at about 10.5 h, when the third peak appears. For the two rest mixtures with SF addition, CSA0.3-SF+FA and CSA0.3-SF+FA+SL, the accumulative heat starts to increase after the second peak at an earlier age compared to that of CSA0.3-SF. To summarize, the addition of SF can obviously increase the hydration heat of the CSA paste at an age around 0.7 to 1.3 h. The heat evolution at the end of the tests (40 h after mixing) decreases with the increase of SCMs in an approximately proportional manner. Based on the present tests, the hydration heat at 40 h after mixing of the CSA pastes (w/c = 0.3) with different use of SCMs is 202.7, 195.2 and 196.2, 191.7, 179.8 J/g, respectively, for CSA0.3 (0% SCMs), CSA0.3-FA and CSA0.3-SF (10% SCMs), CSA0.3-SF+FA (20% SCMs), CSA0.3-SF+FA+SL (30% SCMs).

#### 3.2.3. Early Hydration Heat of the CSA Paste with Different w/c

**Figure 9:**
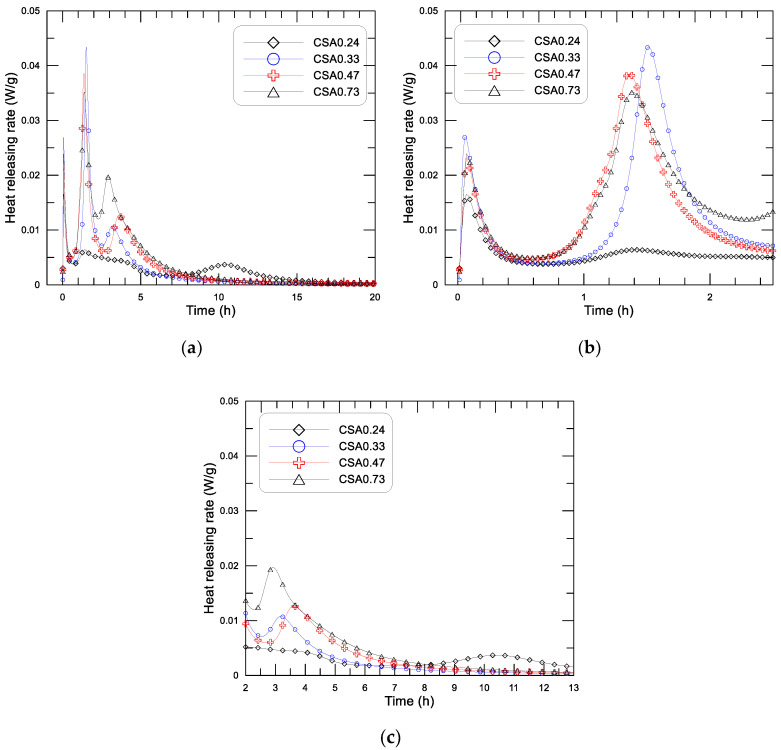
Heat-releasing rate of the CSA paste with different w/c: (**a**) results of 20 h, (**b**) results of the initial 2.5 h, (**c**) results of 2–13 h.

**Figure 10:**
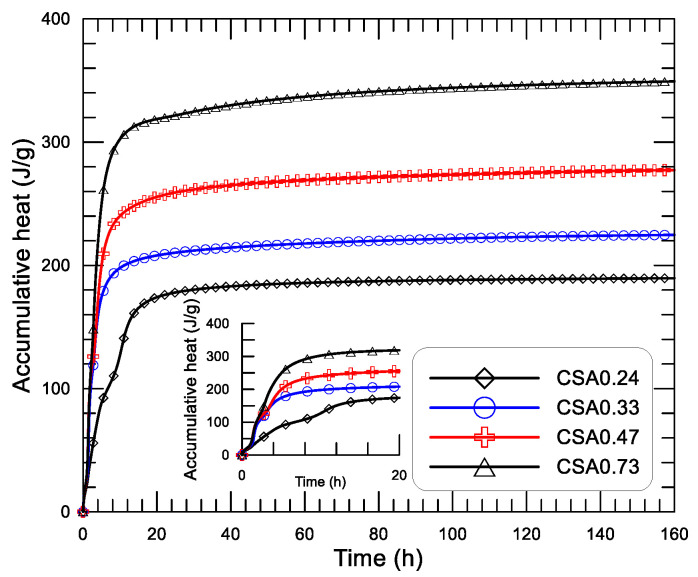
Accumulative heat-age diagrams of CSA pastes with different of w/c.

Figure 9 displays the diagrams of the releasing rate of hydration heat and age since mixing of the four CSA pastes with water to cement ratio (w/c) of 0.24, 0.33, 0.47, and 0.73. Figure 9a shows the results within 20 h, Figure 7b presents the results within the initial 2.5 h, and Figure 9c shows the results of 2 to 13 h. The diagram of accumulative heat and age (up to 160 h) of the pastes with different w/c is presented in Figure 10, in which the inserted small figure displays the results at the initial 20 h.

From Figure 9a, first, the three exothermic peaks that are noted in both the OPC and CSA pastes with w/c of 0.3 can still be observed on the pastes with w/c varying from 0.24 to 0.73. The analyses of the formation mechanism of each exothermic peak were already discussed in the previous section. Second, Figure 9b shows that the dissolution heat peak of the pastes can be divided into two groups, 0.0166 W/g and 0.0269, 0.0231, and 0.0239 W/g, respectively, for CSA0.24 and CSA0.33, CSA0.47, and CSA0.73. For a given particle composition, the dissolution heat should be a constant as long as water can be well adsorbed on the surface of the particles. For the pastes with w/c of 0.33, 0.47, and 0.73, the water supplied may already be sufficient for the adsorption of the cement particles, while the water in the paste with w/c of 0.24 may not be sufficient for cement adsorption and may partially be adsorbed by the superplasticizer used in the paste. Therefore, the dissolution heat peak of CSA0.24 is a little lower than that of the rest of the pastes. No effect of w/c on the appearing time of the dissolution heat peak is observed. Third, a similar duration of the induction period is noted in the pastes with different w/c, ended at around 1 h after mixing. The magnitude of the second exothermic peak (first reaction peak) in the acceleration period is significantly influenced by w/c. The value of the second peak is 0.0064, 0.0434, 0.0386, and 0.0352 W/g, respectively, for CSA0.24, CSA0.33, CSA0.47, and CSA0.73. Obviously, a much lower value of the second heat flow peak is noted for the paste with w/c of 0.24, while a comparable value of the second peak is noted for the rest pastes with w/c of 0.33, 0.47, and 0.73. These findings indicate, for the CSA paste within the initial 2 h after mixing, that the hydration heat is greatly influenced as w/c changes from 0.33 to 0.24, while it may not be influenced significantly as w/c varies from 0.33 to 0.73. The third exothermic peak (second reaction peak) in the deceleration period is also greatly influenced by w/c (see Figure 9c). According to the appearing time of the third peak, the mixtures can also be divided into two groups: group one consists of CSA0.33, CSA0.47, and CSA0.73, and the occurring time of the third peak is around 3–4 h, and group two consists of CSA0.24, and the occurring time of the third peak is 10.5 h. The value of the third maxima of heat flow is 0.0037, 0.0114, 0.0126, and 0.0197 W/g, respectively, for the pastes with w/c of 0.24, 0.33, 0.47, and 0.73. Apparently, the third exothermic peak gradually decreased with the decrease of w/c from 0.73 to 0.33, and dramatically decreases as w/c changes from 0.33 to 0.24, apart from the obvious difference on the appearing time of the third heat flow peak. This may indicate that the ion diffusion in the paste is significantly influenced as w/c is changed from 0.33 to 0.24, while the influence is more gradual as w/c is changed from 0.33 to 0.73. The effect of w/c on the microstructure of the paste should be responsible for the above changes either on the peak strength or on the appearing time of the third heat flow peak. Certainly, the critical value of w/c for the above changes of the CSA paste may not be exactly equal to 0.24 (they should be between 0.33 to 0.24), but it is guaranteed as w/c is less than 0.24. 

From Figure 10, the accumulative heat and age diagrams of the CSA pastes with different w/c can also be divided into two groups within 2 h (first and second exothermic peak controlled period). Group one consist of the low heat-releasing paste of CSA0.24 (low second peak) only, and the other group consists of CSA0.33, CSA0.47, and CSA0.73 (relative higher second peak, which presents a relative higher accumulative heat. After 2 h, the accumulative heat starts to develop under different rates of CSA0.33, CSA0.47, and CSA0.73 (third exothermic peak controlled period). In this stage, for a given age, the larger the w/c, the higher the accumulative heat. The accumulative heat of CSA0.24 starts to grow at a later age due to the delayed appearing of the third peak (see Figure 9c). At about 20 h after mixing, the accumulative heat-age diagram steps into a stage under a low and gradually slower rate. Clearly, the accumulative heat decreases with the decrease of w/c in a later age (after 2 h). At the end of the tests, 160 h later, the accumulative heat is 189.6, 224.7, 277.4, and 349.3 J/g, respectively, for CSA0.24, CSA0.33, CSA0.47, and CSA0.73. It should be noted that although the higher hydration heat means a higher amount of hydration products or a higher cement hydration degree, the strength or the mechanical properties of the cement paste is not simply governed by the amount of hydration products or the cement’s hydration degree. The strength or other mechanical properties of cement paste or concrete is proportional to the volume of capillary pores in the paste, which is normally in reverse proportion to w/c. Therefore, the compressive strength of the CSA pastes with different w/c increases significantly with the decrease of w/c (see the test results listed in Table 2). 

#### 3.2.4. Combination Effect of w/c and SCMs on Early Hydration Heat

**Figure 11:**
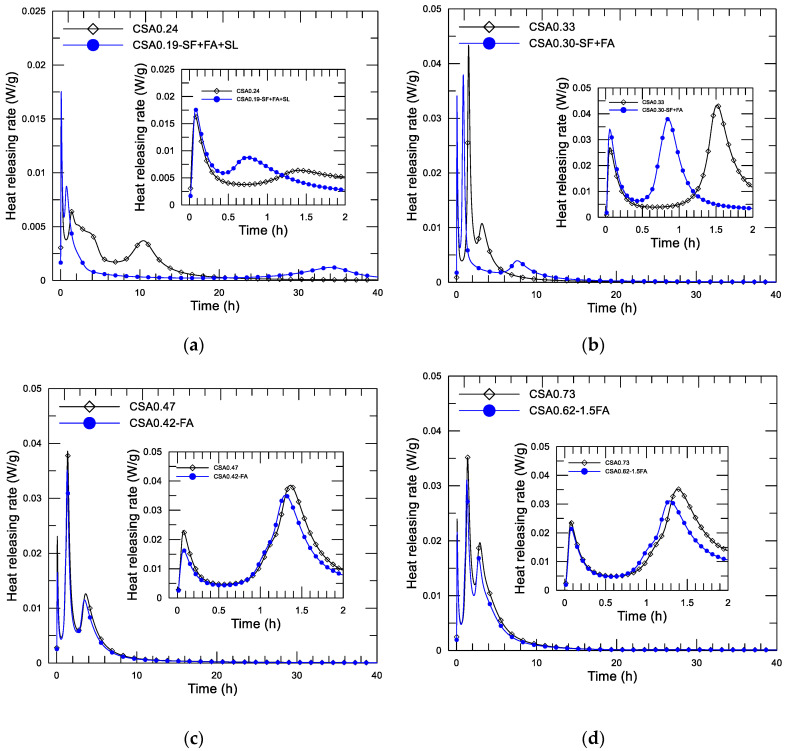
Heat-releasing rate of CSA pastes with effect of addition of SCMs and w/c, (**a**) CSA0.24, CSA0.19-SF+FA+SL, (**b**) CSA0.33, CSA0.30-SF+FA, (**c**) CSA0.47, CSA0.42-FA, (**d**) CSA0.73, CSA0.62-1.5FA.

**Figure 12:**
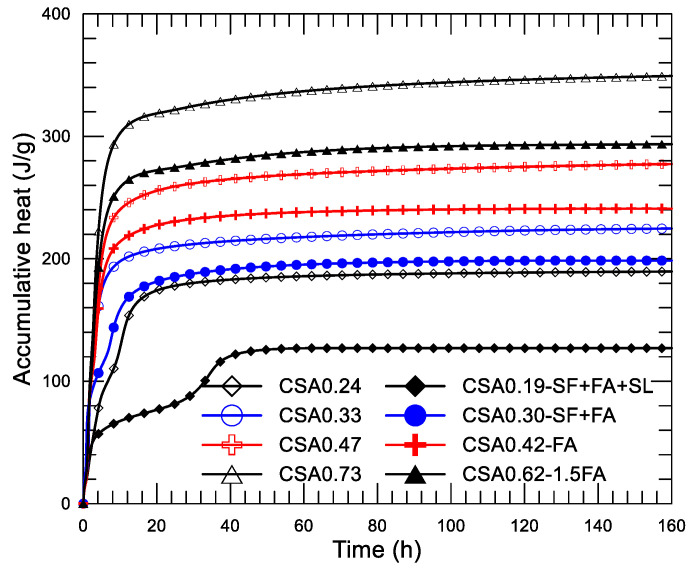
Accumulative heat-age diagrams of CSA pastes with different of w/c and SCMs.

As described in the section on mixture design, the fourth series was intended to study the combined effect of water to binder ratio and SCMs on the hydration heat of the CSA paste. In this series, four pairs of mixtures were formed according to the water to binder ratio, and each pair of mixtures may be used in concrete to form different strength grades in practice. The four pairs of pastes are (CSA0.24, CSA0.19-SF+FA+SL), (CSA0.33, CSA0.30-SF+FA), (CSA0.47, CSA0.42-FA), and (CSA0.73, CSA0.62-1.5FA). Figure 11a–d display the diagram of the heat-releasing rate and age since the mixing of the four pairs of the CSA pastes, respectively. The inserted small figure in each shows the result of the initial 2 h to present the results more clearly. Corresponding accumulative heat-age diagrams (up to 160 h) of the pastes are presented in Figure 12.

First, Figure 9a,b offer a double confirmation of the action of SF, i.e., advancing the first reaction peak (from 1.5 h of CSA0.24 and CSA0.33 to about 0.8 h of CSA0.19-SF+FA+SL and CSA0.30-SF+FA) and delaying the second reaction peak (from 10.5 h of CSA0.24 and 3.5 h of CSA0.33 to about 35 h of CSA0.19-SF+FA+SL and 7.5 h of CSA0.30-SF+FA). The action of nucleation and consolidation of SF in the paste is obvious. Second, the effect of w/c on the heat-releasing characteristic of the CSA paste is confirmed again. The first reaction peak of CSA0.19-SF+FA+SL is comparable to that of CSA0.24. A similar observation can also be found on CSA0.30-SF+FA and CSA0.33. The second reaction peak is significantly delayed for CSA0.19-SF+FA+SL compared to CSA0.30-SF+FA due to the lower w/c, leading to slow ion diffusion. Figure 11c,d show that the addition of FA hardly influences the releasing heat of the paste. A few advances and a little reduction on the second (first reaction peak) and third (second reaction peak) exothermic peaks, compared with the reference mixture, are observed. They may be simply due to the weak nucleation, dilution, and filler actions. It should be noted that the difference on w/c in each pair of mixtures plays a role in the process of heat releasing.

**Figure 13:**
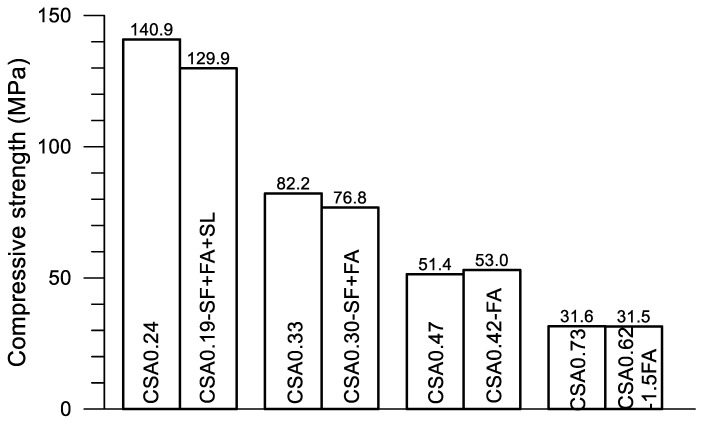
Compressive strength of the four pairs of CSA pastes.

**Figure 14:**
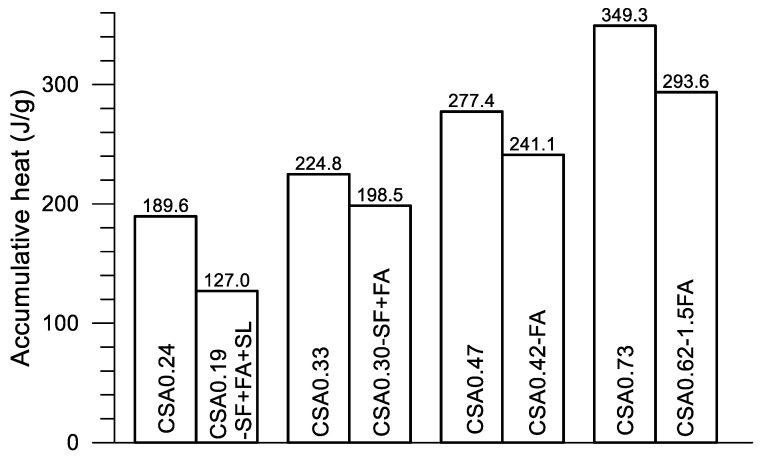
Hydration heat at 160 h of the four pairs of CSA pastes.

In Figure 12, great differences on the progress of accumulative heat can be observed among the four pairs of mixtures. Accumulative heat is reduced with the decrease of w/c and the addition of SCMs under a given age. As described previously, each pair of mixtures is designed for a given strength grade of concrete. Using the compressive strength data given in Table 2, Figure 13 presents the strength at 28 days of the four pairs of pastes. The accumulative heat at 160 h after the mixing of the corresponding paste is displayed in Figure 14. Clearly, four strength grades—130–140, 80–75, 50–53 and 30–32 MPa—are obtained using the present four pairs of paste mixtures, although a certain amount of SCMs are used in the mixtures. On the other hand, the accumulative heat is significantly reduced after using SCMs in the mixture. For CSA0.24 and CSA0.19-SF+FA+SL, the accumulative heat at 160 h is reduced by 33.0% after the use of SCMs (30% in weight), while the compressive strength is reduced only 7.8%. Certainly, saving cement in CSA0.19-SF+FA+SL is also an advantage of the mixture. Similarly, for CSA0.33 and CSA0.30-SF+FA, the accumulative heat is reduced by 11.7% after the use of SCMs (20% in weight), while the compressive strength is reduced by 6.6%. For CSA0.47 and CSA0.42-FA, the accumulative heat is reduced by 13.1% after the use of SCMs (10% in weight), while the compressive strength is increased by 3.1%. For CSA0.73 and CSA0.62-1.5FA, the accumulative heat is reduced by 15.9% after the use of SCMs (15% in weight), while the compressive strength is reduced only by 0.3%. Through an optimized use of SCMs in the CSA paste, both strength and low hydration heat are guaranteed, apart from the significant improvements on the rheological properties of fresh CSA pastes with SCMs [10,26].

## 4. Conclusions

The conclusions based on the present study are as follows:

1. Compared with OPC, the main hydration products of the CSA paste at 3 days are AFt and Al(OH)_3_, and almost no Ca(OH)_2_ is observed; a few AFm may exist at 3 days.
2. Three exothermic peaks were observed—dissolution exothermic peak and two reaction exothermic peaks—under the same w/c (w/c = 0.3), the first and second reaction peaks of the CSA paste are 17.8 times and 4.1 times higher that of the OPC paste, and the occurring time is much earlier than that of the OPC paste.
3. Compared with FA, SF can significantly promote the formation of AFt at an early age and delay its formation locally at a later age; the second reaction peak appears earlier, and the third reaction peak appears later in the pastes with addition of SF than in those without SF.
4. A decrease of w/c can greatly decrease the two reaction exothermic peaks of the CSA paste, and it seems that there is a critical value of w/c or w/b, which should be located between 0.24 and 0.30; above the critical value, the effect of w/c is minor, and below that the influence it is obvious.
5. An optimal use of SCMs in the CSA pastes with different w/c can greatly decrease the releasing heat while maintaining the required strength.

## Figures and Tables

**Table 1:** Chemical composition and physical properties of the cementitious materials.

No.	Chemical Composition (wt.%)									Apparent Density (g/cm^3^)	Surface Area (m^2^/kg)
	SiO_2_	CaO	Al_2_O_3_	Fe_2_O_3_	MgO	K_2_O	Na_2_O	SO_3_	LOI		
OPC	25.77	55.55	6.15	4.46	3.72	0.76	0.21	3.09	1.49	3.14	356
CSA	6.19	42.9	24.16	1.04	1.96	0.48	0.08	12.91	8.91	2.82	423
SF	90.56	0.81	0.41	0.52	0.95	1.59	0.63	–	3.72	2.11	20,000
FA	47.02	5.08	35.06	3.88	1.36	1.30	1.18	0.89	1.85	2.30	540
SL	38.83	38.70	12.92	1.46	4.63	0.37	0.28	0.60	0.06	2.78	550

**Table 2:** Mixture proportion, fluidity, and compressive strength of the cement pastes.

No.	Notation	Cement	SCMs	Water	PC (wt.%)	SD (mm)	Compressive Strength (MPa)	
							3d	28d
1	OPC0.3	1	0	0.3	0.2	260	62.3	81.5
2	CSA0.3	1	0	0.3	0.2	250	76.6	86.4
3	CSA0.3-FA	0.9	0.1(FA)	0.3	0.2	270	57.3	72.5
4	CSA0.3-SF	0.9	0.1(SF)	0.3	0.2	220	68.6	83.4
5	CSA0.3-SF+FA	0.8	0.1(SF) + 0.1(FA)	0.3	0.2	230	58.5	76.8
6	CSA0.3-SF+FA+SL	0.7	0.1(SF) + 0.1(FA) + 0.1(SL)	0.3	0.2	255	42.5	70.5
7	CSA0.33	1	0	0.33	0.2	260	73.1	82.2
8	CSA0.24	1	0	0.24	0.8	255	120.3	140.9
9	CSA0.19-SF+FA+SL	0.7	0.1(SF) + 0.1(FA) + 0.1(SL)	0.19	0.8	210	108.3	129.9
10	CSA0.42-FA	0.9	0.1(FA)	0.42	0.05	220	42.7	53.0
11	CSA0.47	1	0	0.47	0.05	230	45.3	51.4
12	CSA0.62-1.5FA	0.85	0.15(FA)	0.62	0	250	25.5	31.5
13	CSA0.73	1	0	0.73	0	270	26.7	31.6

## Data Availability

The data presented in this study are available on request from the corresponding author.
